# Sulfonation-Time-Dependent Structure–Property Relationships of Electrospun Polyketone Nanofiber Membranes for PEMFC Applications

**DOI:** 10.3390/polym18121542

**Published:** 2026-06-21

**Authors:** Hongsik Byun, Geon-Hyeong Lee, Yeol-Lim Lee, Sang-Hun Lee

**Affiliations:** 1 Department of Chemical Engineering, Keimyung University, Daegu 42601, Republic of Korea; ecocatlgh@gmail.com (G.-H.L.); yllee@kmu.ac.kr (Y.-L.L.); 2 Department of Environmental Engineering, Keimyung University, Daegu 42601, Republic of Korea

**Keywords:** polyketone, electrospinning, sulfonation time, nanofiber membrane, ion exchange capacity, water uptake, structure–property relationship, proton conductivity, PEMFC (Polymer Electrolyte Membrane Fuel Cell)

## Abstract

Electrospun sulfonated polyketone (PK) nanofiber membranes were prepared to investigate the sulfonation-time-dependent structure–property relationships of hydrocarbon-based polymer electrolyte membranes for PEMFC (Polymer Electrolyte Membrane Fuel Cell) applications. NaCl addition to the electrospinning solution increased solution conductivity and enabled the formation of uniform PK nanofibers with an average diameter of approximately 270 nm. Subsequent sulfonation introduced sulfonic-acid-related groups into the PK nanofiber framework, and the resulting membrane properties were strongly governed by sulfonation time. Among the tested membranes, PK-NC16 exhibited the highest proton conductivity of 0.107 ± 0.031 S cm^−1^ and an ion exchange capacity of 2.82 m_eq_ g^−1^, exceeding or comparable to those of Nafion 115 under the tested conditions. FTIR-based analysis indicated that the relative sulfonation index increased up to 16 h, whereas extended sulfonation for 24 h generated additional sulfone/sulfonate-related bands, suggesting possible side reactions or structural changes under prolonged acid treatment. The high water uptake of PK-NC16 enhanced proton transport but also revealed a hydration-sensitive polymer network, as reflected by a voltage degradation rate of approximately −590 μV h^−1^ during a 100 h short-term stability constant-current test. These results demonstrate that sulfonation time is a key parameter controlling the balance among ionic functionality, hydration, mechanical response, proton conductivity, and PEMFC-relevant single-cell performance in electrospun PK nanofiber membranes.

## 1. Introduction

Proton-exchange membrane fuel cells (PEMFCs) have attracted considerable attention as efficient electrochemical energy-conversion devices because they directly convert the chemical energy of hydrogen into electricity through electrochemical oxidation and reduction reactions [1,2,3]. In a typical PEMFC, hydrogen is oxidized at the anode to generate protons and electrons, while oxygen reduction occurs at the cathode to produce water. The proton-exchange membrane located between the electrodes plays a central role in this process by transporting protons from the anode to the cathode while physically separating the fuel and oxidant gases. Therefore, the membrane strongly influences not only the ohmic resistance of the cell, but also gas crossover, water management, mechanical integrity, and long-term durability.

Perfluorosulfonic acid (PFSA) membranes such as Nafion™ have been widely used as benchmark PEM materials because of their high proton conductivity and chemical stability under hydrated conditions. PFSA membranes have an amphiphilic molecular architecture composed of a chemically robust hydrophobic perfluorinated backbone and hydrophilic sulfonic-acid side chains. Under hydrated conditions, the phase separation between the fluorinated matrix and ionic sulfonic-acid clusters generates interconnected proton-conducting pathways, which accounts for the high proton conductivity, oxidative stability, and mechanical integrity of Nafion-type benchmark membranes. However, their relatively high cost, complex manufacturing process, and hydration-dependent dimensional changes remain important limitations for broader commercialization. In particular, repeated swelling and shrinkage during fuel-cell operation can induce mechanical stress, interfacial instability between the membrane and catalyst layers, and performance degradation over extended operation. These limitations have motivated extensive research into hydrocarbon-based proton-conducting membranes as lower-cost and chemically tunable alternatives to fluorinated membranes [4,5,6,7,8].

Sulfonated hydrocarbon polymers are attractive PEM candidates because proton-conducting acid groups can be introduced into hydrocarbon polymer frameworks, allowing the ionic functionality, water uptake, and mechanical properties to be adjusted through chemical modification. Representative examples include sulfonated poly(ether sulfone), poly(ether ketone), polyimide, and poly(phenylene sulfide)-based membranes [4,5,6,7]. These materials can exhibit high ion-exchange capacity and proton conductivity, but they often suffer from excessive water uptake, dimensional instability, and insufficient durability under practical fuel-cell operating conditions [8,9,10]. Therefore, the development of hydrocarbon-based PEMs requires not only high proton conductivity, but also a balanced control of hydration, mechanical stability, and membrane–electrode compatibility. Nevertheless, it was reported that the recent progress in sulfonated hydrocarbon membranes, particularly sulfonated poly(ether ether ketone) (SPEEK) and SPEEK-based composite membranes, has further demonstrated that hydrocarbon PEMs can be tuned to balance proton conductivity, cost, and mechanical stability through control of sulfonation degree and composite architecture [11].

Polyketone is a promising hydrocarbon polymer platform because of its chemical stability, relatively low material cost, and processability into fibrous membrane structures. In particular, electrospinning provides a versatile route for fabricating micro- and nanofiber membranes with controllable fiber diameter, pore structure, and surface morphology [12]. Nanofibrous architectures can provide large surface areas and interconnected pathways that may be beneficial for subsequent chemical modification and proton transport. However, when polyketone-based nanofiber membranes are considered for PEMFC applications, the extent of sulfonation must be carefully controlled. Insufficient sulfonation may result in low proton conductivity, whereas excessive sulfonation can induce high water uptake, structural instability, or side reactions that reduce the effective transport performance of the membrane.

In this study, electrospun polyketone nanofiber membranes were prepared using a NaCl-assisted electrospinning process and subsequently sulfonated for different reaction times. The primary objective was to clarify the sulfonation-time-dependent structure–property relationships of PK nanofiber membranes, with particular emphasis on chemical modification, nanofiber morphology, ion exchange capacity, hydration behavior, mechanical response, and proton conductivity. Single-cell PEMFC tests, including OCV retention, polarization behavior, and short-term constant-current operation, were further conducted to evaluate whether the optimized polymer membrane properties could be translated into electrochemical performance. This work therefore provides a polymer-materials perspective on the potential and limitations of sulfonated PK nanofiber membranes as hydrocarbon-based polymer electrolyte membrane candidates.

## 2. Experimental

### 2.1. Materials

Polyketone (PK, Mw = 180,000 g/mol, Hyosung Corporation, Seoul, Republic of Korea) was employed as the base polymer for electrospinning. 1,1,1,3,3,3-hexafluoro-2-propanol (HFIP, Daejung Chemical & Metals Co., Ltd., Gyeonggi-do, Republic of Korea) and methanol (Daejung Chemical & Metals Co., Ltd.) were used as solvents. Sodium chloride (NaCl, Duksan Pure Chemical Co., Ltd., Gyeonggi-do, Republic of Korea) was used as an additive, and sodium borohydride (NaBH_4_, Daejung Chemical & Metals Co., Ltd.) was used as a membrane reducing agent. n-Hexane (Duksan Pure Chemical Co., Ltd.) and acetone (Daejung Chemical & Metals Co., Ltd.) were used for membrane washing after reduction. All chemicals were used without separate purification. Sulfonation was performed to convert the PK nanofiber membrane into a conductive membrane; for this purpose, concentrated sulfuric acid (99%) was prepared as 30 wt% sulfuric acid using distilled water without purification and used. The PK used in this study is an aliphatic carbon monoxide–olefin copolymer containing repeating ketone groups, which can be schematically represented as –[–CH2–CH(R)–CO–]n–, where R denotes hydrogen or an alkyl substituent depending on the olefin unit. In the present modification route, NaBH_4_ treatment partially reduces ketone groups to hydroxyl-containing structures, whereas sulfuric-acid treatment introduces sulfonic-acid/sulfonate-related functionalities into the PK nanofiber framework.

### 2.2. PK Electrospinning and Sulfonation

**Figure 1:**
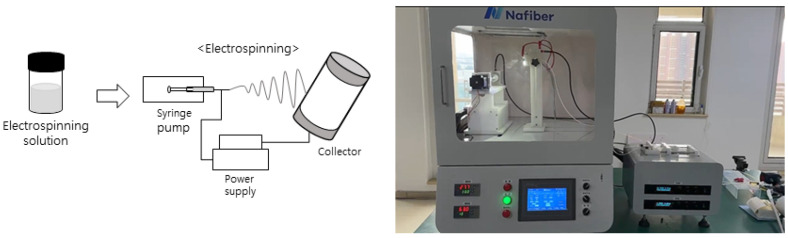
The diagram of electrospinning equipment and its picture.

The schematic diagram of the electrospinning process and the actual equipment used are shown in Figure 1. The procedure was conducted as follows. First, the PK polymer was added to either a single solvent of HFIP or a mixed solvent of HFIP, methanol, and sodium chloride. The mixture was stirred at 180 rpm for 12 h to ensure complete dissolution. The composition ratios of the polymer solutions used in this study are summarized in Table 1.

The prepared electrospinning solution was loaded into a 5 mL syringe and kept vertically for at least 30 min to completely remove any residual air bubbles. To ensure stable voltage supply and proper Taylor cone formation, a stainless steel needle (tip) was attached to the front of the syringe, which was then mounted on a syringe pump (KDS100, KD Scientific Inc., Seoul, Republic of Korea). Electrospinning was performed by connecting a DC high-voltage generator (LT100Pro, Shandong Nafeibo Technology Development Co., Ltd., Shandong, China) to the tip.

The injection rate of the PK solution was maintained at 0.8 mL/h using the syringe pump, and needle gauges of 26 G and 30 G were utilized. The rotation speed of the collector roller was kept at 480 mm/s using a universal collecting drum controller. Voltage optimization was conducted to prevent the solution from scattering due to excessive voltage or failing to overcome surface tension due to insufficient voltage. The final optimized voltage and spinning process parameters are listed in Table 2.

Electrospinning of solutions without salt additives was carried out under two different environments: low humidity and high humidity, with humidity levels controlled using a humidifier. The membrane fabricated under low humidity was designated as PK-LH, while the one produced under high humidity was named PK-HH. To increase electrical conductivity and produce thinner fibers, salt was added to the spinning solution, and the needle was replaced with a finer 30 G gauge. The membrane produced under these optimized conditions was designated as PK-NaCl.

After spinning, the membranes were dried in a vacuum oven at 40 °C for 24 h to remove residual solvents. The dried membranes were then post-processed into 8-layered structures using a heating press (DHP-2, Dae Heung Science, Incheon, Republic of Korea) according to the conditions in Table 3, followed by characterization.

To enhance the hydrophilicity of the final PK-NaCl nanofiber membrane, a 0.5 wt% NaBH_4_ aqueous solution was permeated through the membrane at 0.3 bar for 30 s using a vacuum pump. The resulting reduced membrane was named rPK-NaCl. During this reduction process, extreme care was taken to minimize the swelling of the nanofibers. The reduced membranes were washed sequentially with D.I. water, acetone, and n-hexane, and then dried at room temperature [12]. Finally, the dried membranes underwent an additional processing step according to the conditions in Table 3 to minimize any potential deformation.

The final nanofiber membranes were sulfonated using diluted sulfuric acid (30.0 wt%) with continuous stirring. Severe sulfonation conditions may promote side reactions or crosslink-like structural changes in sulfonated polymer membranes. To minimize such undesired structural changes, sulfonation in this experiment was carried out at 30 °C. A large excess of sulfuric acid (~700 mL of 30 wt% sulfuric acid/~0.1 g of dry nanofiber membrane) was used to remove the water formed in the sulfonation step. Various reaction times with sulfuric acid (8, 16 and 24 h) were employed, and the extent of modification for each treatment was assessed by various methods as listed below.

After sulfonation, the porous sulfonated electrospun mats were converted into pressed proton-conducting membranes by the post-treatment pressing process before electrochemical and fuel-cell-relevant measurements. This clarification is important because the precursor mat morphology and the final membrane morphology can differ substantially after densification. For clarity, the term electrospun mat is used in this manuscript to describe the porous fibrous precursor obtained after electrospinning and chemical treatment, whereas the term pressed proton-conducting membrane refers to the densified membrane obtained after hot pressing and used for IEC, proton conductivity, water uptake, mechanical testing, and MEA evaluation.

### 2.3. Characterizations

#### 2.3.1. Pore Characterization

The mean pore size of the PK nanofiber membranes was measured using a PMI Automated Perm-porometer (Porous Materials, Inc., Ithaca, NY, USA). The capillary flow porometry method was employed for the analysis, using Pore-wick with a surface tension of 16 dynes/cm as the wetting liquid. Detailed measurement conditions for the pore size analysis are summarized in Table 4 below. Pore size and porosity were measured using the electrospun mat state to evaluate the precursor fibrous network before complete densification.

#### 2.3.2. Porosity Measurement

For porosity measurement, a dried PK nanofiber membrane was cut into 5 × 5 cm (width × length) pieces, its thickness was measured to calculate its volume, and the weighed membrane was immersed in n-butanol (Duksan Pure Chemicals Co., Ltd.) for 2 h. After removing the membrane, the n-butanol on the surface was removed using filter paper. The weight was then measured and substituted into the following Formula (1) to calculate the porosity.

(1) $$P\;\left(\%\right)=\;\frac{W_{\omega }-W_{\delta }}{\rho_{b}V_{p}}$$

$W_{d}$: Weight of dry membrane

$W_{\omega }$: Weight of membrane impregnated with n-butanol

$\rho_{b}$: Density of n-butanol

$V_{p}$: Volume of dry membrane

#### 2.3.3. Ion Exchange Capacity and Proton Conductivity Measurements

Ion exchange capacity is one of the electrochemically important characteristics. Through this, the extent of inclusion of hydrophilic cation exchange groups within the prepared membrane can be determined. The ion exchange capacity was measured using the following experimental method. First, the sulfonated PK nanofiber membrane was immersed in 1N NaOH for 4 h, then immersed in 1N HCl for 12 h to replace the form of the sulfonated copolymer terminal groups, and then dried in an oven. Then, it was immersed again in 0.5N NaOH for 12 h, titrated with a 0.01M NaOH solution, and the ion exchange capacity was calculated by substituting into Equation (2). IEC and proton conductivity measurements were performed using pressed sulfonated membranes rather than the loose electrospun mats.

IEC = (VNaOH × CNaOH)/mdry(2)

where VNaOH = Volume of NaOH used, CNaOH = Concentration (N) of NaOH used, mdry = Weight of dry membrane

Proton conductivity was determined by cutting the sample into 4 cm × 4 cm pieces, drying them thoroughly in a vacuum oven, and then using an impedance analyzer (WEIS). Complex impedance was measured in the frequency range from 10 Hz to 1 MHz using stainless steel electrodes. The sample measurements were repeated 5 times and the average value was calculated. The DC resistance was determined as the value when the Z’ phase was 0. Using the DC resistance obtained in this way, the proton conductivity was calculated using the following Equation (3) [13].

(3) $$\sigma (S/\mathrm{cm})=\frac{L(\mathrm{cm})}{R\left(\Omega \right)\times W\left(\mathrm{cm}\right)\times d(\mathrm{cm})}$$

where σ = proton conductivity, L = length of the membrane, R = direct current resistance, W = width of the membrane, d = distance of the electrode

#### 2.3.4. Water Uptake, Dimensional Change and Mechanical Properties Measurements

To evaluate the stability of the membranes under fuel cell operating conditions, the water uptake, dimensional change (area) and mechanical properties, i.e., tensile strength and elongation were measured. For water uptake and dimensional change measurements, five membranes (50 × 50 mm) were prepared and immersed in 80 °C distilled water for 24 h. After the water uptake, the residual water from the sample’s surface was removed by the absorption paper. The water uptake and dimensional change of the membranes were then recorded and calculated using the following Equations (4) and (5), respectively. Water uptake, dimensional change and mechanical properties were measured using the pressed membranes to represent the state used for MEA fabrication.

(4) $$Water\;uptake\;\left(\%\right)=\frac{W_{wet}-W_{dry}}{W_{dry}}$$

where W_wet_ = weight of fully water absorbed membrane, W_dry_ = weight of dried membrane.

(5) $$Dimentional\;change\;\left(\%\right)=\frac{A_{wet}-A_{dry}}{A_{dry}}$$

where A_wet_ = area of fully water absorbed membrane, A_dry_ = area of dried membrane.

The tensile strength and elongation of the final sulfonated PK nanofiber membranes were measured using a KYUNG-SUNG Instron Testing Machine (Gyeonggi-do, Republic of Korea) in accordance with the ASTM D882 standard [14]. Specimens were prepared with dimensions of 10 cm × 3 cm. The tests were conducted at a crosshead speed of 500 mm/min, and each sample was measured three times for comparative analysis and to ensure reproducibility.

#### 2.3.5. Polarization Curve Test (IV Test) and Open Circuit Voltage Test (OCV Test)

Open-circuit voltage (OCV) and current-voltage (I-V) tests were conducted using a fuel cell test system (Fuel Cell Power Co., Ltd., Republic of Korea). In general, the OCV test is an essential procedure performed to identify hydrogen crossover caused by membrane defects such as pinholes, or to detect faulty MEAs and gas leaks.

For these tests, the pressed PK nanofiber membranes were used and MEAs were fabricated by Fuel Cell Power Co., Ltd. Although the specific details of the fabrication process are proprietary, the general procedure is as follows: first, a uniform catalyst ink (slurry) is prepared by mixing a platinum (Pt) catalyst, conductive additives, and an ion-conductive polymer binder in a solvent. This catalyst ink is then uniformly coated onto the electrolyte membrane using a doctor blade method. After coating, the solvent is removed through a drying process, leaving only the catalyst layer. Finally, a gas diffusion layer (GDL) is placed on top of the catalyst layer and pressed under high temperature and pressure to produce an integrated MEA [15].

**Figure 2:**
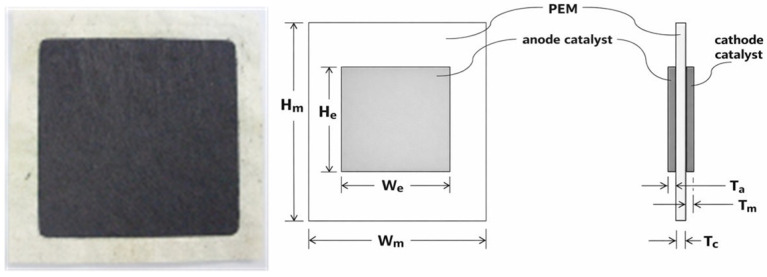
Photo of MEA and its schematic diagram.

The actual appearance and a simplified schematic of the fabricated MEA are shown in Figure 2. The specific conditions for the single-cell and I-V tests are summarized in Table 5 and the active area of the GDE/MEA used for the single-cell test was 25 cm^2^.

## 3. Results and Discussion

### 3.1. Formation of Electrospun Polyketone Nanofiber Membranes

**Figure 3:**
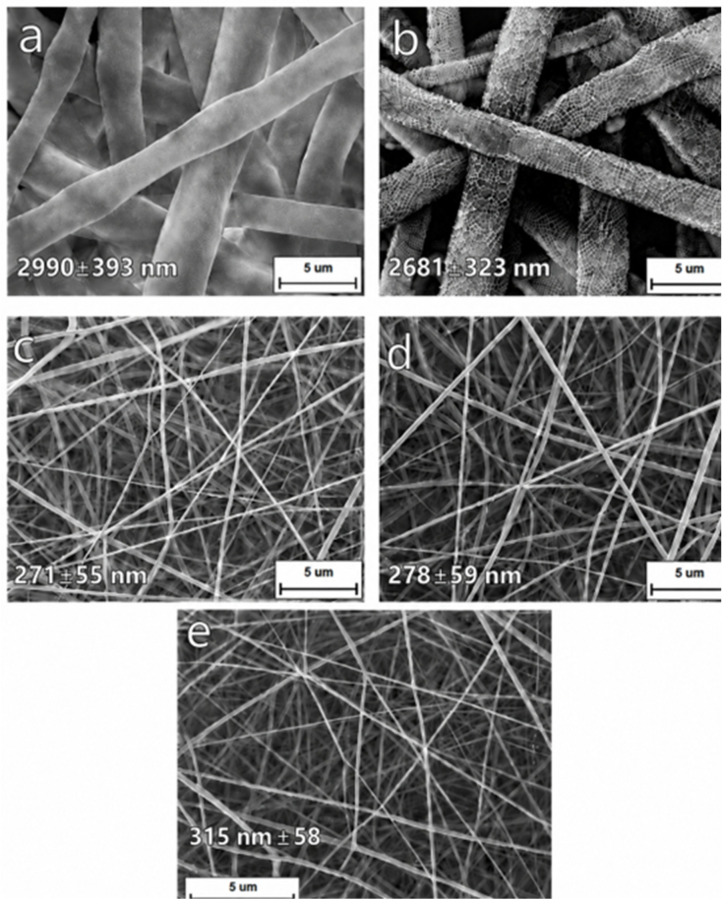
SEM images (×5000) and fiber diameters of PK micro/nano fiber membranes; (**a**) PK-LH, (**b**) PK-HH, (**c**) PK-NaCl, (**d**) rPK-NaCl, (**e**) pressed PK-NaCl.

The morphology of the electrospun polyketone membranes was strongly affected by the composition and electrical conductivity of the spinning solution. Figure 3 shows the SEM images and fiber-diameter distributions of the PK-based micro/nanofiber membranes prepared under different electrospinning conditions. When PK was electrospun from the HFIP solution without an ionic additive, the resulting fibers were in the micrometer range. The average fiber diameters of PK-LH and PK-HH were approximately 2681 and 2990 nm, respectively, indicating that humidity control alone was insufficient to produce nanoscale PK fibers under the present conditions.

In contrast, the addition of NaCl to the electrospinning solution markedly reduced the fiber diameter. As summarized in Table 6, the electrical resistance of the PK solution decreased from 11.5 MΩ for PK-LH to 2.5 MΩ for PK-NaCl, while the solution conductivity increased from 0.266 to 1.224 μS cm^−1^. This increase in solution conductivity is expected to enhance charge repulsion within the polymer jet and promote stretching during electrospinning, thereby producing thinner fibers [16,17,18]. As a result, PK-NaCl exhibited uniform nanofibers with an average diameter of approximately 270 nm. This confirms that the NaCl-assisted electrospinning approach was effective for converting PK fibers from the micrometer scale to the nanometer scale. The change in fiber diameter also affected the pore structure of the membranes. As shown in Table 7, the average pore size decreased from the micrometer range for PK-LH and PK-HH to approximately 0.2 μm for PK-NaCl and rPK-NaCl. This reduction can be attributed to the denser packing of thinner nanofibers, which decreases the inter-fiber void size. The porosity of PK-NaCl remained high, indicating that the membrane retained a porous nanofibrous architecture despite the substantial decrease in pore size. Such a structure is advantageous for subsequent chemical modification because it provides a large surface area and facilitates the penetration of the sulfonating medium into the fibrous membrane network.

**Figure 4:**
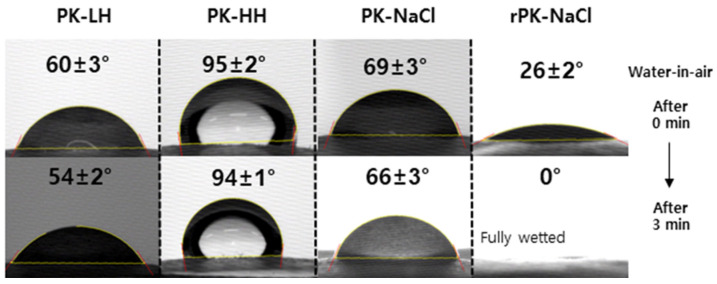
Contact angle measurement of PK micro/nano fiber membranes.

**Figure 5:**
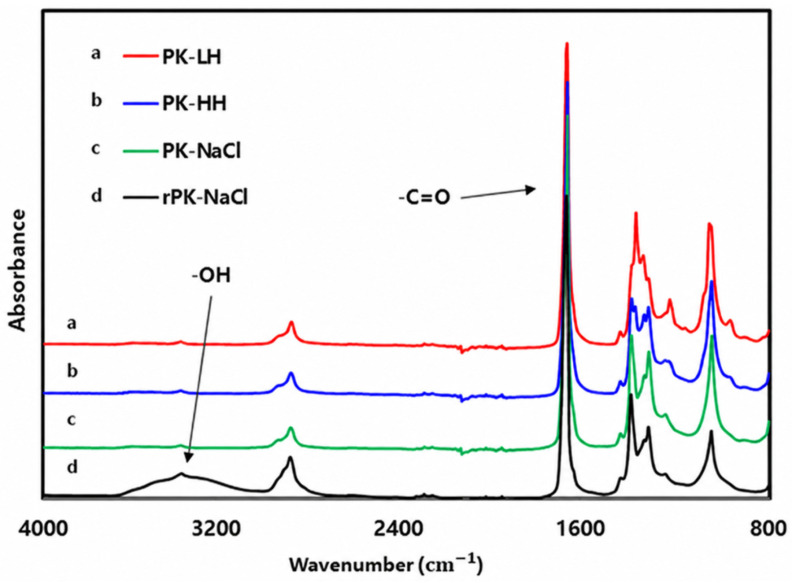
FTIR spectra of PK micro/nanofiber membranes.

The NaBH_4_-treated rPK-NaCl membrane showed increased hydrophilicity, as indicated by the contact-angle results in Figure 4. FTIR analysis further supported the chemical change induced by reduction treatment. As shown in Figure 5, the characteristic carbonyl stretching band of PK appeared at approximately 1690 cm^−1^, together with CH_2_-related bands at approximately 1411 and 1335 cm^−1^. After NaBH_4_ treatment, a broad band appeared in the 3200–3600 cm^−1^ region, which can be assigned to hydroxyl groups formed by partial reduction of ketone groups. Although increased hydrophilicity may be beneficial for water uptake and ion transport, excessive hydrophilicity may also increase the risk of swelling and dimensional instability under fuel-cell operating conditions. Therefore, PK-NaCl was selected and compressed to produce a major precursor membrane for subsequent sulfonation and PEMFC evaluation. Figure 3e shows the SEM of pressed PK-NaCl nanofiber membrane, indicating that the nanofiber size increased slightly from 271 nm ± 55 to 315 nm ± 58. However, the pore size and porosity did not change significantly, except a 19% decrease in thickness (Table 7). The reduced membrane thickness may be beneficial for lowering internal ohmic resistance, particularly at high current densities. However, excessive thinning can increase the risk of gas crossover and may reduce OCV and fuel efficiency [19].

To compare the relative extent of sulfonation among the tested sulfonation times and to evaluate possible structural changes in PK-NC24, a semi-quantitative FTIR analysis was conducted [20,21,22]. The semi-quantitative estimation was performed by calculating the intensity ratio between the sulfonate stretching band near 1030 cm^−1^ and the ketone C=O stretching band. The 1180 cm^−1^ band was not used for the relative calculation because it may overlap with additional sulfone/sulfonate-related bands in PK-NC24, making direct quantitative comparison unreliable.

The FTIR-based relative sulfonation index followed the order PK-NC16 ≥ PK-NC24 > PK-NC8. Because FTIR alone cannot unambiguously determine the detailed chemical structure of the additional sulfur-containing species, the new bands observed for PK-NC24 should be interpreted as evidence of possible side reactions or crosslink-like structural changes rather than as conclusive proof of a specific sulfone-bridge structure. Further XPS, solid-state NMR, and elemental sulfur analysis will be required to identify the exact chemical structures formed during prolonged sulfonation. Adding to that, because only three sulfonation times were investigated in this work, PK-NC16 should be regarded as the most favorable condition among the tested 8, 16, and 24 h samples rather than as a universally optimized sulfonation time. Additional intermediate conditions such as 12 and 20 h will be required to define a narrower optimum sulfonation window.

### 3.2. Chemical Modification of PK Nanofiber Membranes by Sulfonation

**Figure 6:**
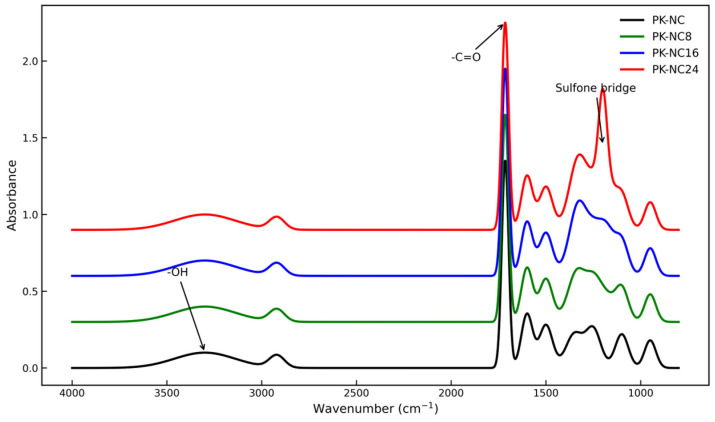
FTIR spectra of sulfonated PK-NC membranes.

To introduce proton-conducting acid groups into the PK nanofiber membrane, PK-NaCl membranes were sulfonated in diluted sulfuric acid for different reaction times. The resulting membranes were designated as PK-NC8, PK-NC16, and PK-NC24 according to the sulfonation time of 8, 16, and 24 h, respectively. Figure 6 presents the FTIR spectra of the non-sulfonated and sulfonated PK-NC membranes.

After sulfonation, new absorption bands appeared in the region associated with sulfonic-acid-related groups. In particular, bands near 1030 and 1180 cm^−1^, together with a shoulder around 1120 cm^−1^, can be assigned to S=O stretching and related vibrations of sulfonic acid or sulfonate groups. These spectral changes indicate that acid-functional groups were introduced into the PK nanofiber membranes during the sulfonation process. The intensity of these bands increased with sulfonation time up to 16 h, suggesting that the density of proton-conducting functional groups increased under the intermediate sulfonation condition.

For PK-NC24, additional bands appeared in the region around 1320 and 1150 cm^−1^. These bands may be associated with additional sulfone/sulfonate-related structures generated under prolonged acid treatment. However, because FTIR alone cannot definitively determine the detailed chemical structure or crosslinking configuration, these bands should be interpreted as evidence of possible side reactions or crosslink-like structural changes rather than conclusive proof of a specific sulfone-bridge structure. This conservative interpretation is important because excessive sulfonation can alter the polymer network in ways that do not necessarily improve proton transport. This interpretation is consistent with recent SPEEK studies showing that excessive sulfonation can increase water uptake and swelling while reducing mechanical durability and operational stability [22]. Therefore, the 24 h condition was interpreted not as a simple increase in useful acid functionality but as a prolonged acid-treatment condition that may introduce competing structural effects.

To compare the relative extent of sulfonation among the membranes, a FTIR-based relative sulfonation index was estimated from the baseline-corrected intensity ratio between the sulfonic-acid-related band and the carbonyl band of the PK backbone. The carbonyl band was used as an internal reference because it originates from the PK framework and remained clearly observable after sulfonation. The relative intensity ratio was expressed as:

R = A_SO3_/A_C=O_ (6)

where A_SO3_ is the baseline-corrected intensity of the sulfonic-acid-related band and A_C=O_ is the baseline-corrected intensity of the carbonyl band. The detailed calculation procedure is provided in the Supplementary Materials [23,24].

The FTIR-based relative sulfonation index followed the order PK-NC16 ≥ PK-NC24 > PK-NC8. The approximate intensity ratios were estimated to be ~0.6 for PK-NC8, ~1.1 for PK-NC16, and ~0.9 for PK-NC24. Although this trend indicates that the 16 h sulfonation condition produced the highest relative sulfonation level among the tested samples, this index was obtained from baseline-corrected FTIR peak ratios and should therefore be interpreted as a semi-quantitative indicator rather than an absolute degree of sulfonation. Absolute DS determination would require additional elemental analysis, sulfur content quantification, or independent acid-base titration; therefore, the present FTIR analysis was used only to support the relative trend among the sulfonation times [25,26,27].

Notably, despite the longer reaction time, PK-NC24 did not show a further increase in the relative sulfonation index compared with PK-NC16. This result suggests that prolonged sulfonation did not simply increase the number of effective proton-conducting groups. Instead, extended acid treatment may have promoted side reactions, structural rearrangement, or partial network rigidification, which could reduce the effective accessibility of acid groups and disturb proton-conducting pathways. Therefore, the 16 h sulfonation condition appears to provide a more favorable balance between acid-functional-group introduction and preservation of the membrane structure.

### 3.3. Water Uptake. Dimensional Changes and Mechanical Properties

Hydration behavior is a key polymer property of sulfonated nanofiber membranes because it directly affects ionic-domain formation, proton mobility, dimensional stability, and mechanical response. However, excessive water uptake can also lead to dimensional instability, swelling, mechanical weakening, and poor membrane–electrode interfacial stability during fuel-cell operation. Therefore, water uptake should be interpreted not only as an indicator of proton-conducting hydration channels, but also as a potential source of durability problems.

**Figure 7:**
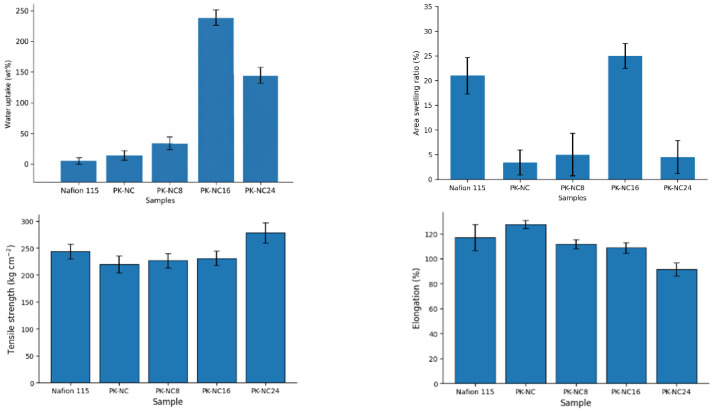
Water uptake, dimensional change (area) and mechanical properties of sulfonated PK-NC.

Figure 7 and Table 8 show the water uptake and area swelling ratio of Nafion 115 and the PK-NC membranes. Nafion 115 exhibited a water uptake of 18.9 ± 5.2 wt%, whereas the non-sulfonated PK-NC membrane showed a higher value of 36.6 ± 8.4 wt%. After sulfonation, the water uptake increased substantially. PK-NC8 showed a water uptake of 55.9 ± 9.6 wt%, while PK-NC16 exhibited the highest value of 243.8 ± 10.3 wt%. This large increase is attributed to the introduction of hydrophilic acid groups, which promote water absorption and the formation of hydrated ionic domains. The high water uptake of PK-NC16 is consistent with its high IEC and proton conductivity, as discussed in Section 3.4.

However, the extremely high water uptake of PK-NC16 also indicates a potential limitation. Although a highly hydrated membrane environment can facilitate proton transport, excessive water absorption may induce swelling, morphological changes, and interfacial stress between the membrane and catalyst layer during fuel-cell operation. Therefore, the water uptake of PK-NC16 should be regarded as both a beneficial factor for proton conduction and a possible cause of durability degradation. On the other hand, the swelling ratios of PK-NC samples were found to be very stable compared to commercial Nafion 115 except PK-NC16. Although PK-NC16 showed a high swelling ratio, it did not show significantly higher swelling ratio compared to Nafion 115. However, it can be expected that the high swelling ratio of PK-NC16 may play an important role in the low durability.

PK-NC24 showed a lower water uptake of 160.5 ± 9.7 wt% than PK-NC16, despite the longer sulfonation time. This decrease is consistent with the FTIR results, which suggested that prolonged sulfonation may induce additional sulfone/sulfonate-related side reactions or crosslink-like structural changes. Such structural changes can restrict polymer-chain mobility and reduce the ability of the membrane to absorb water. Therefore, the water uptake trend supports the conclusion that excessive sulfonation does not necessarily improve the membrane hydration environment or proton transport properties.

The tensile strength and elongation results are also shown in Figure 7 and Table 8. Nafion 115 exhibited a tensile strength of 243.3 ± 13.9 kg cm^−2^ and an elongation of 117.0 ± 10.7%. The PK-based membranes showed tensile strengths in a comparable range, with PK-NC, PK-NC8, and PK-NC16 exhibiting values of 219.4 ± 15.7, 226.9 ± 13.2, and 231.2 ± 13.1 kg cm^−2^, respectively. PK-NC24 showed the highest tensile strength of 278.2 ± 18.6 kg cm^−2^, together with the lowest elongation of 91.5 ± 5.3%. This behavior suggests that prolonged sulfonation may increase membrane rigidity, possibly due to structural rearrangement or crosslink-like effects under extended acid treatment. Overall, the mechanical-property results indicate that the PK-NC membranes retained mechanical integrity after sulfonation, but the balance between hydration and rigidity was strongly dependent on sulfonation time.

### 3.4. Ion Exchange Capacity and Proton Conductivity

**Figure 8:**
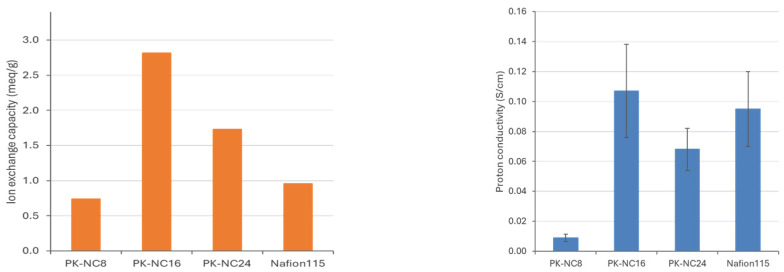
IEC and proton conductivity of sulfonated PK-NC and Nafion 115.

The ion exchange capacity and proton conductivity of the sulfonated PK-NC membranes are shown in Figure 8 and Table 9. Both properties were strongly influenced by sulfonation time. PK-NC8 exhibited a relatively low IEC of 0.74 meq g^−1^ and a proton conductivity of 0.0089 ± 0.0025 S cm^−1^. These values indicate that 8 h sulfonation was insufficient to generate a sufficient density of proton-conducting acid groups and interconnected ionic pathways.

PK-NC16 showed the highest IEC and proton conductivity among the tested membranes. The IEC reached 2.82 meq g^−1^, and the proton conductivity was 0.107 ± 0.031 S cm^−1^. Under the present measurement conditions, this conductivity was comparable to or slightly higher than that of Nafion 115, which showed a proton conductivity of 0.095 ± 0.025 S cm^−1^. This result indicates that the 16 h sulfonation condition produced a highly hydrated and proton-conductive membrane environment. The high conductivity of PK-NC16 is consistent with its high water uptake and the relatively high FTIR-based sulfonation index. Although the high proton conductivity of PK-NC16 is correlated with high IEC and water uptake, the present data do not allow a definitive separation of Grotthuss-type proton hopping and vehicle-type proton transport. Temperature-dependent conductivity measurements and activation-energy analysis, together with nanoscale ion-domain characterization, will be required to identify the dominant conduction mechanism.

However, the high IEC of PK-NC16 should not be interpreted only as a positive feature. A high density of acid groups can enhance proton transport, but it can also increase water uptake and swelling. Therefore, the performance of PK-NC16 reflects a trade-off between high proton conductivity and hydration-sensitive dimensional stability. This trade-off is particularly important for practical PEMFC operation, where membrane hydration changes continuously with gas flow, current density, temperature, and relative humidity.

PK-NC24 exhibited an IEC of 1.73 meq g^−1^ and a proton conductivity of 0.068 ± 0.014 S cm^−1^. Although these values were higher than those of PK-NC8, they were lower than those of PK-NC16. This indicates that extending the sulfonation time from 16 to 24 h did not further improve the effective proton transport properties. The reduced conductivity of PK-NC24 may be associated with the formation of less accessible acid groups, side reactions, or structural rigidification under prolonged acid treatment. These effects can reduce the continuity of proton-conducting pathways even when additional sulfur-containing groups are present.

Overall, the IEC and conductivity results demonstrate that proton transport in the sulfonated PK-NC membranes is governed by a balance among acid-group density, water uptake, and structural accessibility. Among the tested samples, PK-NC16 provided the most favorable balance, resulting in the highest proton conductivity and IEC. Therefore, PK-NC16 was selected as the representative membrane for further single-cell PEMFC evaluation.

From a polymer-materials perspective, these results demonstrate that the performance of sulfonated PK nanofiber membranes is not determined solely by the amount of acid functionality. Instead, proton transport is governed by the coupled effects of sulfonation level, water uptake, polymer-chain mobility, and nanofiber morphology [28,29,30,31,32,33,34]. PK-NC8 contained insufficient proton-conducting sites, resulting in low IEC and proton conductivity. PK-NC24, despite the longer sulfonation time, did not show further improvement, likely because prolonged acid treatment generated additional sulfone/sulfonate-related structures and increased membrane rigidity. PK-NC16 provided the most favorable balance among acid functionality, hydration, and structural accessibility, leading to the highest proton conductivity. This trend highlights the importance of optimizing chemical modification conditions rather than simply maximizing sulfonation time. The present results demonstrate the usefulness of the electrospun nanofiber architecture for chemical modification and membrane design; however, a direct comparison with dense cast PK films at matched IEC and thickness was not included. Such a control will be required in future work to quantify the intrinsic advantage of the nanofibrous structure relative to conventional casting.

### 3.5. Open-Circuit Voltage Retention and Polarization Performance

To examine whether the optimized polymer membrane properties could be translated into PEMFC-relevant performance, the sulfonated PK-NC membranes were evaluated in single-cell tests. In this context, OCV retention and polarization behavior were used as practical validation tests for the membrane properties, rather than as standalone indicators of complete fuel-cell durability [35]. OCV retention provides an indirect indication of gas-barrier integrity and MEA stability, but it can also be affected by electrode condition, gas sealing, and operating stability.

**Figure 9:**
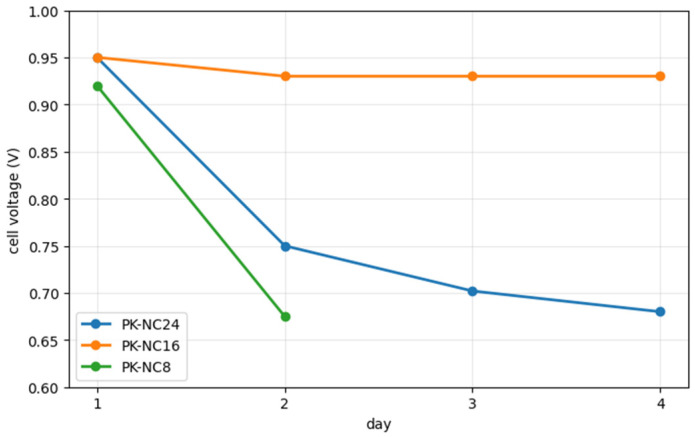
OCV test results of sulfonated PK-NC.

Figure 9 shows the OCV behavior of PK-NC8, PK-NC16, and PK-NC24 over time. PK-NC16 exhibited the most stable OCV among the tested membranes, maintaining a voltage of approximately 0.93–0.95 V over 4 days. This stable OCV suggests that the PK-NC16 membrane retained sufficient gas-barrier integrity and MEA stability under open-circuit conditions. This behavior is consistent with the high proton conductivity and balanced membrane properties observed for PK-NC16, because proton transport in sulfonated hydrocarbon PEMs is generally governed by the coupled effects of acid-group density, hydration, morphology, and ionic-domain connectivity [30,31,32,33,34,35].

In contrast, PK-NC8 showed rapid OCV decay, decreasing from approximately 0.92 V to 0.675 V within the first 2 days. This poor OCV retention may be related to insufficient sulfonation and the resulting poor proton-conducting network, which can lead to unstable membrane hydration and poor electrochemical performance. PK-NC24 also exhibited a continuous OCV decrease from approximately 0.95 V on day 1 to 0.68 V on day 4. Although PK-NC24 had higher IEC and conductivity than PK-NC8, its inferior OCV stability compared with PK-NC16 suggests that excessive sulfonation may have adversely affected membrane structure or MEA stability through side reactions or crosslink-like structural changes under prolonged acid treatment [36,37,38]. These results support the conclusion that the optimal sulfonation condition should provide not only sufficient acid functionality, but also a stable membrane structure suitable for fuel-cell operation.

**Figure 10:**
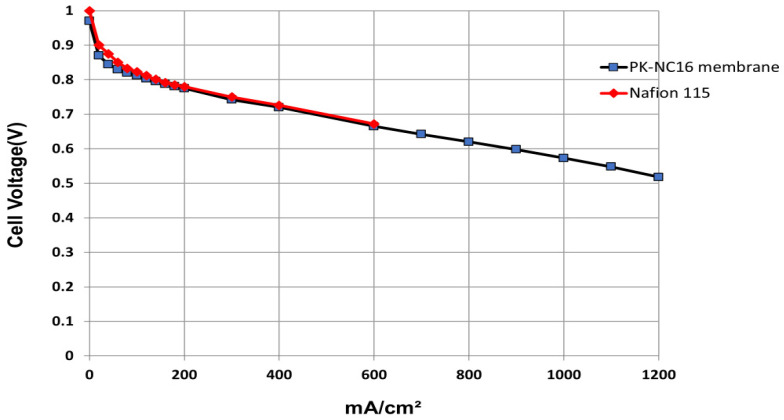
IV test of Nafion 115 and PK-NC16.

Based on the OCV results, PK-NC16 was selected for further polarization and durability tests. Figure 10 compares the polarization behavior of the PK-NC16 membrane with that of Nafion 115 under the tested single-cell conditions. The PK-NC16 MEA showed polarization behavior comparable to that of the Nafion 115 MEA in the intermediate current-density region. This result indicates that the high proton conductivity of PK-NC16 was effectively reflected in the single-cell performance.

To obtain a semi-quantitative comparison of the ohmic-loss tendency [39], the apparent area-specific resistance was estimated from the slope of the intermediate quasi-linear region of the polarization curves between 200 and 600 mA cm^−2^. The apparent resistance was approximately 0.274 Ω cm^2^ for PK-NC16 and 0.270 Ω cm^2^ for Nafion 115. The difference between these values was small and likely within the uncertainty of graphical estimation. Therefore, the PK-NC16 membrane exhibited an apparent ohmic-polarization behavior comparable to that of Nafion 115 under the selected operating conditions.

However, this value should not be interpreted as a direct high-frequency resistance. The slope of a DC polarization curve includes activation, ohmic, and mass-transport contributions simultaneously [40]. Therefore, the estimated value should be described as an apparent area-specific resistance, R_app_, rather than as a direct HFR. Accurate separation of membrane resistance from kinetic and mass-transport losses requires electrochemical impedance spectroscopy, high-frequency AC resistance measurement, or current-interrupt analysis [41,42,43,44]. Nevertheless, the polarization results demonstrate that PK-NC16 can function as a proton-conducting membrane in a PEMFC single cell and can provide short-term performance comparable to Nafion 115 in the tested current-density range.

### 3.6. Short-Term Constant-Current Durability and Remaining Limitations

**Figure 11:**
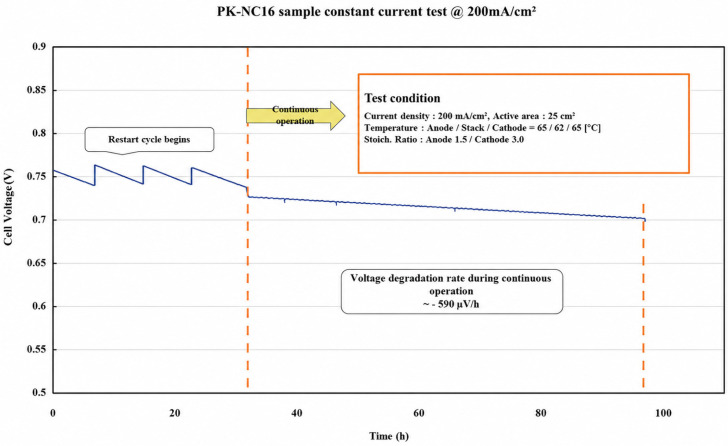
Constant current test of PK-NC16.

To further evaluate the operational stability of PK-NC16, a short-term constant-current durability test was performed at 200 mA cm^−2^ for 100 h. The test was conducted under H_2_/air operation, and the cell voltage profile is shown in Figure 11. During the initial period from 0 to approximately 32 h, the cell voltage exhibited periodic fluctuations associated with restart cycles. Each restart produced a transient voltage recovery followed by gradual voltage decay. This behavior may be attributed to temporary rehydration of the membrane, redistribution or removal of liquid water, and recovery of local mass-transport conditions during restart operation.

After approximately 32 h, the cell entered a more continuous operating regime. During this period, the voltage decreased nearly linearly from approximately 0.73 to 0.70 V. The corresponding voltage degradation rate was approximately −590 μV h^−1^. This degradation rate indicates that, although PK-NC16 exhibited promising initial performance and stable OCV retention, its short-term operational stability still requires improvement.

The voltage decay observed during constant-current operation is likely related to the hydration-sensitive nature of the PK-NC16 membrane, although PEMFC voltage loss can generally involve coupled contributions from membrane dehydration, water-management instability, interfacial degradation, catalyst-layer degradation, and mass-transport limitations [39,45]. As discussed above, PK-NC16 exhibited very high water uptake of 243.8 ± 10.3 wt%. While such high hydration is beneficial for proton conduction, it may also make the membrane highly sensitive to changes in gas flow, relative humidity, and water balance during fuel-cell operation. Repeated hydration and dehydration can induce morphological changes, swelling–shrinkage stress, and interfacial instability between the membrane and catalyst layers. These effects can increase ohmic resistance and accelerate voltage loss. In addition, defects, incomplete densification, or stratification generated during pressing of the sulfonated electrospun mats may contribute to interfacial resistance and local delamination. Therefore, optimization of the pressing conditions and direct cross-sectional morphology analysis of the MEA membrane are necessary in future work.

However, the degradation mechanism cannot be assigned solely to membrane resistance based on the present data. Catalyst-layer degradation, loss of electrochemically active surface area, changes in ionomer distribution, gas-diffusion limitations, and interfacial contact resistance may also contribute to the voltage decay. Therefore, additional in situ and post-mortem diagnostics are required to identify the dominant degradation mechanism. In particular, electrochemical impedance spectroscopy during constant-current operation would allow direct tracking of high-frequency resistance and charge-transfer resistance. Hydrogen crossover measurements would clarify gas-barrier stability, while dimensional swelling tests and post-operation SEM or FTIR analysis would provide information on membrane morphology and chemical stability after fuel-cell operation. Start–stop cycling, low-humidity operation, open-circuit voltage cycling, Fenton-type chemical aging, and AST-based durability protocols should also be performed in future work to evaluate practical PEMFC applicability.

Overall, the single-cell results support the structure–property interpretation derived from the membrane characterization data. PK-NC16 achieved the most favorable combination of acid functionality, hydration, proton conductivity, and short-term OCV stability among the tested membranes. However, its high water uptake and measurable voltage decay during the 100 h constant-current test indicate that the optimized proton-conducting network was accompanied by hydration-sensitive dimensional and interfacial instability. Therefore, from a polymer design perspective, future work should focus on controlling the sulfonation degree while suppressing excessive swelling, for example through controlled crosslinking, composite reinforcement, optimized nanofiber densification, or post-sulfonation stabilization. Direct electrochemical diagnostics such as in situ EIS, together with dimensional swelling and post-mortem morphology analyses, will be required to connect polymer structural changes with long-term PEMFC durability.

## 4. Conclusions

Electrospun sulfonated polyketone nanofiber membranes were prepared to investigate the sulfonation-time-dependent structure–property relationships of hydrocarbon-based polymer electrolyte membranes for PEMFC applications. NaCl-assisted electrospinning increased the solution conductivity and enabled the formation of uniform PK nanofibers with an average diameter of approximately 270 nm. Subsequent sulfonation introduced sulfonic-acid-related groups into the PK nanofiber framework, and the resulting membrane properties were strongly governed by sulfonation time.

Among the tested membranes, PK-NC16 exhibited the most favorable balance of proton-conducting functionality and single-cell performance. It showed the highest proton conductivity of 0.107 ± 0.031 S cm^−1^ and an IEC of 2.82 m_eq_ g^−1^, which were comparable to or higher than those of Nafion 115 under the present measurement conditions. FTIR-based analysis indicated that the relative sulfonation index increased up to 16 h, whereas prolonged sulfonation for 24 h did not further improve the effective sulfonation level and generated additional sulfone/sulfonate-related bands, suggesting possible side reactions or structural changes under extended acid treatment. Although the present study identifies PK-NC16 as the most favorable sample among the tested membranes, additional intermediate sulfonation times such as 12 and 20 h will be required to define the optimum sulfonation window more precisely.

The relationship among FTIR-based sulfonation index, IEC, water uptake, mechanical properties, and proton conductivity shows that proton transport in sulfonated PK nanofiber membranes is controlled by a coupled polymer structure–property balance rather than by acid-group density alone. PK-NC8 was insufficiently functionalized, whereas PK-NC24 appeared to suffer from reduced structural accessibility and increased rigidity after prolonged acid treatment. PK-NC16 provided the best compromise, but its high water uptake of 243.8 ± 10.3 wt% also revealed the hydration-sensitive nature of the polymer network.

PEMFC single-cell tests further demonstrated that the optimized polymer membrane properties of PK-NC16 could be translated into practical electrochemical performance, as shown by stable OCV retention of approximately 0.93–0.95 V over 4 days and polarization behavior comparable to Nafion 115 in the intermediate current-density region. However, the 100 h constant-current test revealed a voltage degradation rate of approximately −590 μV h^−1^, indicating that swelling control and hydration management remain critical for improving durability.

Overall, this study demonstrates that the performance of sulfonated PK nanofibrous membranes is governed not simply by increasing sulfonation time, but by the synergistic regulation of NaCl-assisted nanofiber formation and controlled sulfonation. Compared with conventional sulfonated hydrocarbon PEM approaches, the PK nanofibrous platform provides a processable architecture for balancing proton conductivity and mechanical integrity, although further durability improvement remains necessary. Future work should focus on reducing excessive water uptake while preserving high proton conductivity through controlled sulfonation, crosslinking or reinforcement strategies, dimensional stabilization, and direct in situ diagnostics such as EIS and hydrogen crossover measurements.

In summary, the main innovation of this work is the synergistic regulation of NaCl-assisted nanofiber formation and sulfonation time, which enables simultaneous control of fiber morphology, acid functionality, hydration behavior, mechanical response, and PEMFC-relevant performance. The results demonstrated that sulfonated PK nanofiber membranes were promising proof-of-concept hydrocarbon-based PEM candidates, but they were not yet direct practical replacement for Nafion-type membranes. However, future work may also explore composite or hybrid strategies, including approaches inspired by SPEEK-based membranes, to improve the durability of PK-based nanofibrous PEMs while retaining their high proton conductivity.

## Figures and Tables

**Table 1:** Composition of polyketone electrospinning solutions (LH: Low humidity (30%), HH: High humidity (70%)).

Samples	PK (g)	HFIP (g)	Methanol (g)	NaCl (g)
PK-LH	0.88	10.12	-	-
PK-HH	0.88	10.12	-	-
PK-NaCl	0.531	7.61	0.846	0.009

**Table 2:** Electrospinning conditions of corresponding PK solutions (TCD: Tip to collector distance).

Samples	Flow Rate (mL/h)	Duration (hr)	Relative Humidity (%)	Syringe Tip (Gauge)
PK-LH	0.8	6	30	26
PK-HH	0.8	6	70	26
PK-NaCl	0.8	6	30	30

**Table 3:** Post-treatment process conditions of corresponding PK membranes.

Samples	Pressure (psi)	Temperature (°C)	Duration (s)
PK-LH	6000	25	5
PK-HH	6000	25	5
PK-NaCl	10,000	40	5

**Table 4:** The measurement conditions for pore size.

	Pressure	Method	Test Gas	Fluid Solution
Pore distribution	Max 50 psi	Capillary Flow Porometer (wet up dry down)	Air	Pore-wick

**Table 5:** The conditions for the single cell and IV tests.

Single Cell test conditions	Catalyst (0.43 mg/cm^2^)	Anode PtRu/C,
		Cathode, Pt/C
	Gas	Anode/Cathode = H_2_ 100%/filtered Air
	Active area	25 cm^2^
IV test conditions	Current	200 mA/cm^2^
	Stoichiometric Ratio (SR)	Anode SR = 1.50
		Cathode SR = 3.00
	Temperature	Stack = 62 °C
		Anode = 65 °C
		Cathode = 65 °C

**Table 6:** Conductivity of PK-LH and PK-NaCl electrospinning solutions.

Samples	Resistance (MΩ)	Conductivity (μS/cm)
PK-LH	11.5	0.266
PK-NaCl	2.5	1.224

**Table 7:** Pore size and porosity of PK micro/nanofiber membranes.

Samples	Biggest Pore Size (μm)	Average Pore Size (μm)	Smallest Pore Size (μm)	Thickness (μm)	Porosity (%)
PK-LH	5.53	2.89	3.65	90 ± 2	82.2 ± 2
PK-HH	3.16	1.43	1.67	88 ± 3	61.1 ± 2
PK-NaCl	0.356	0.192	0.171	58 ± 2	82.6 ± 2
rPK-NaCl	0.376	0.213	0.202	57 ± 3	81.7 ± 3
Pressed PK-NaCl	0.326	0.179	0.148	47 ± 3	79.8 ± 3

**Table 8:** Water uptake, dimensional change (area), mechanical strength of sulfonated PK-NC.

Sample	Water Uptake (%)	
Nafion 115	18.9 ± 5.2	
PK-NC	36.6 ± 8.4	
PK-NC8	55.9 ± 9.6	
PK-NC16	243.8 ± 10.3	
PK-NC24	160.5 ± 9.7	
**Sample**	**Area Swelling Ratio (%)**	**Note**
Nafion 115	21 ± 3.7	Benchmark
PK-NC	3.4 ± 2.5	Pressed membrane
PK-NC8	5 ± 4.3	Pressed membrane
PK-NC16	25 ± 2.5	High water uptake sample
PK-NC24	4.5 ± 3.3	Prolonged sulfonation sample
**Sample**	**Tensile strength (kg/cm^2^)**	**Elongation (%)**
Nafion 115	243.3 ± 13.9	117 ± 10.7
PK-NC	219.4 ± 15.7	127.5 ± 3.2
PK-NC8	226.9 ± 13.2	111.8 ± 3.6
PK-NC16	231.2 ± 13.1	108.7 ± 4.1
PK-NC24	278.2 ± 18.6	91.5 ± 5.3

**Table 9:** Comparison of IEC and proton conductivity of sulfonated PK-NC and Nafion 115.

	PK-NC8	PK-NC16	PK-NC24	Nafion 115
Proton conductivity (S/cm)	0.0089 ± 0.0025	0.107 ± 0.031	0.068 ± 0.014	0.095 ± 0.025
Ion exchange capacity(meq/g)	0.74	2.82	1.73	0.96

## Data Availability

The data presented in this study are available on request from the corresponding author due to confidentially restrictions and proprietary information owned by the industrial partner involved in the experiments.

## Supplementary Materials

The following supporting information can be downloaded at: https://www.mdpi.com/article/10.3390/polym18121542/s1, S1: Schematic representation of PK and proposed chemical modification pathways for NaBH_4_ reduction and sulfonation; S2: Calculation Method for the Relative Intensity Ratio, R; S2-1: Calculation of R for PK-NC8, PK-NC16, and PK-NC24; S3: Interpretation of the OCV test result of PK-NC16 membrane; S4: Indirect HFR Estimation from Polarization Curves (PK-NC16 membrane and Nafion 115 MEAs); S4-1. Calculation Basis; S4-2. Approximate Voltage Values from the Graph; S4-3. Indirect HFR Calculation; S4-4. Interpretation; References [23,24,39,40,41,43,44,45] are cited in Supplementary Materials.
