# Supplementary material for: Sulfonation-Time-Dependent Structure–Property Relationships of Electrospun Polyketone Nanofiber Membranes for PEMFC Applications
Source: Polymers (Basel). 2026 Jun 21;18(12):1542. doi: 10.3390/polym18121542 (PMC13307501; doi:10.3390/polym18121542)
Supplement: Supplementary file 1 [file polymers-18-01542-s001.zip › polymers-4319083-supplementary.pdf]

Supplementary

S1: Schematic representation of PK and proposed chemical modification pathways for $NaBH_4$ reduction and sulfonation.

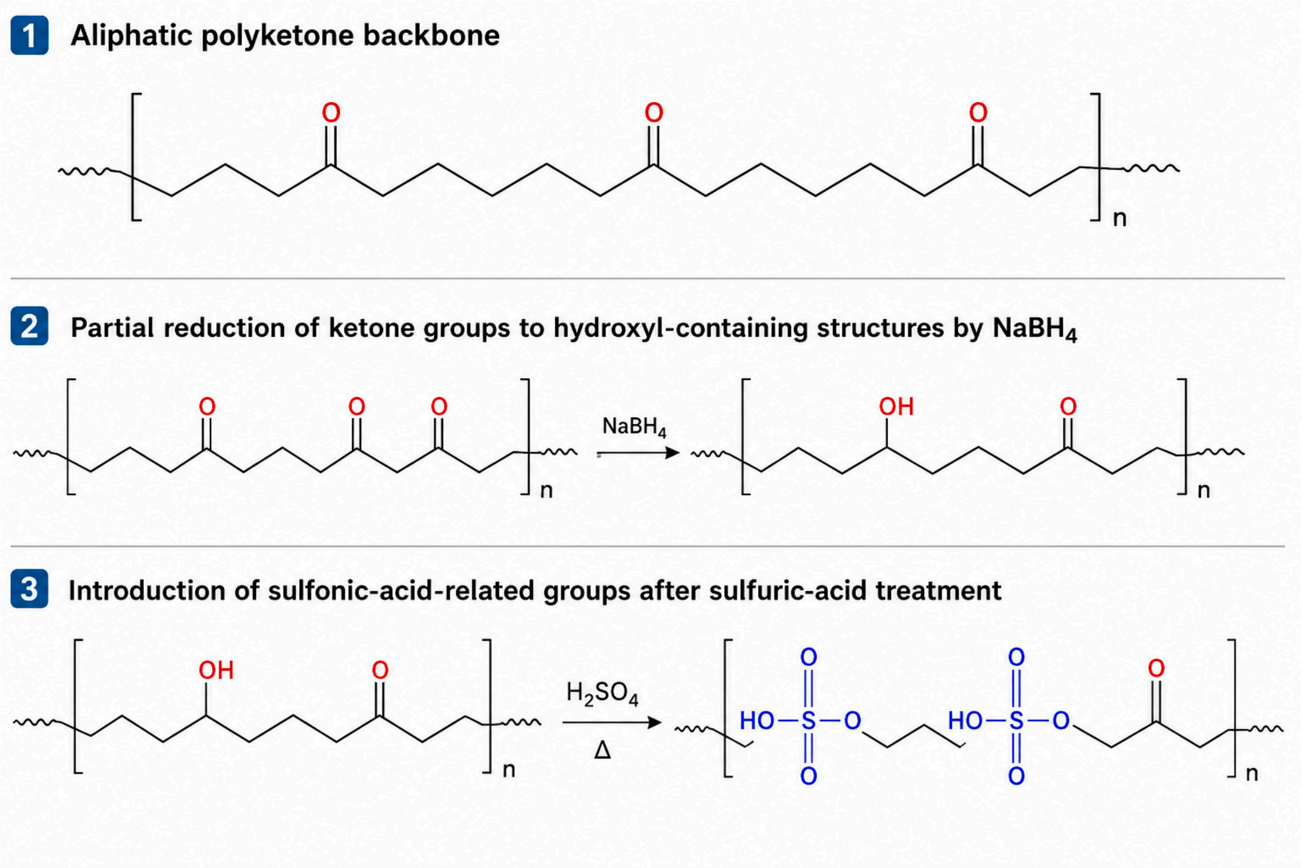

S2: Calculation Method for the Relative Intensity Ratio, R

The relative degree of sulfonation was evaluated using the FT-IR intensity ratio between the sulfonic acid-related peak and the carbonyl peak of the polyketone backbone [1, 2].

The ratio was calculated as follows:

$R = A_{SO3}/A_{C=O}$

where A_{SO3} is the absorbance intensity or peak area of the sulfonic acid-related band, and $A_{C=O}$ is the absorbance intensity or peak area of the carbonyl band.

In this analysis, the carbonyl peak near approximately 1700 cm^{-1} was used as an internal reference because it originates from the polyketone backbone and is expected to remain relatively stable after sulfonation. The sulfonic acid-related peak was assigned to the SO_3H or sulfonate group, typically observed in the region of approximately 1000–1250 cm^{-1}.

Because the FT-IR spectra in the graph are vertically offset for better visibility, the absolute absorbance values shown on the y-axis should not be directly used for the calculation. Instead, each spectrum must first be corrected by subtracting its own baseline.

The calculation procedure is as follows.

First, the baseline of each spectrum is determined. For each sample, the flat region of the spectrum is used as the reference baseline.

Second, the corrected carbonyl peak intensity is obtained by subtracting the baseline value from the carbonyl peak intensity:

$A_{C=O} = I_{C=O,peak} - I_{baseline}$

Third, the corrected sulfonic acid-related peak intensity is obtained in the same way:

$A_{SO3} = I_{SO3,peak} - I_{baseline}$

Finally, the relative intensity ratio is calculated by dividing the corrected SO_3 peak intensity by the corrected C=O peak intensity:

$R = (I_{SO3,peak} - I_{baseline})/(I_{C=O,peak} - I_{baseline})$

Therefore, the R value represents the relative amount of sulfonic acid groups introduced into the polymer structure, normalized by the carbonyl peak of the polyketone backbone.

S2-1: Calculation of R for PK-NC8, PK-NC16, and PK-NC24

Sample	Approx. baseline	Approx. C=O peak intensity	$A_{C=O}$ calculation
PK-NC8	0.30	1.59	1.59 - 0.30 = 1.29
PK-NC16	0.60	1.90	1.90 - 0.60 = 1.30
PK-NC24	0.90	2.19	2.19 - 0.90 = 1.29

The calculated $A_{C=O}$ values are nearly identical, approximately 1.29-1.30. Therefore, the C=O peak can be used as an internal reference for normalizing the SO_3-related band intensity.

Using the reported relative intensity ratios, the SO_3-related baseline-corrected intensity can be back-calculated as follows:

$$A_{SO3} = R \times A_{C=O}$$

Sample	$A_{C=O}$	Reported R	$A_{SO3} = R \times A_{C=O}$	Verification of R
PK-NC8	1.29	0.6	0.6 x 1.29 = 0.774 ≈ 0.77	0.77 / 1.29 ≈ 0.60

PK-NC16	1.30	1.1	1.1 x 1.30 = 1.430 ≈ 1.43	1.43 / 1.30 ≈ 1.10
PK-NC24	1.29	0.9	0.9 x 1.29 = 1.161 ≈ 1.16	1.16 / 1.29 ≈ 0.90

S3: Interpretation of the OCV test result of PK-NC16 membrane

The durability of the PK-NC16 membrane electrode assembly (MEA) was evaluated through a constant current density test at 200 mA/cm² over a period of 100 hours. The operating conditions were maintained at a stack temperature of 62°C with stoichiometric ratios (Stoich.) of 1.5 and 3.0 for the anode and cathode, respectively.

1. Dynamic Behavior during Restart Cycles [3]

In the initial phase of the test (0–32 hours), the cell voltage exhibited a periodic fluctuation characterized by "Restart cycles." Each cycle shows a transient voltage recovery followed by a gradual decline. This phenomenon is typically attributed to the rehydration of the electrolyte membrane and the removal of accumulated liquid water (water flushing) during the restart process, which temporarily reduces the ohmic and mass transport resistances.

2. Voltage Degradation during Continuous Operation [4]

Following the stabilization of the system, a continuous operation mode was initiated at approximately 32 hours. The cell voltage showed a highly linear decay from ~0.73 V to ~0.70 V. The voltage degradation rate was calculated to be -590 μV/h.

This degradation behavior can be analyzed through two primary mechanisms:

Ohmic Resistance Increase: As discussed in previous CV/EIS analyses, the potential for dehydration of the ionomer within the catalyst layer or the membrane itself under specific gas flow conditions can lead to a steady increase in ohmic loss [3].

Electrochemical Surface Area (ECSA) Loss: The continuous operation at 200 mA/cm² may induce carbon support corrosion or Pt catalyst agglomeration, leading to a reduction in the number of active reaction sites [4].

S4: Indirect HFR Estimation from Polarization Curves (PK-NC16 membrane and Nafion 115 MEAs)

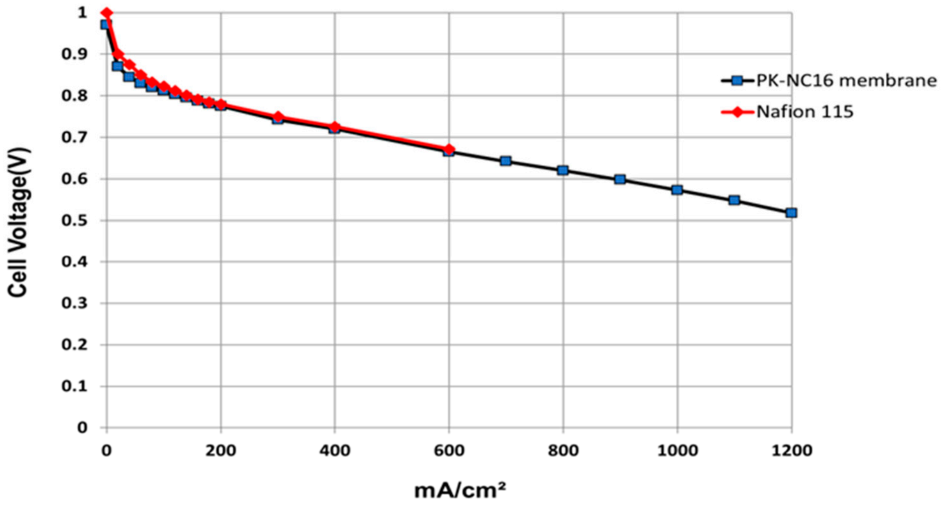

Figure 11. Polarization curves of MEAs employing the PK-NC16 membrane and Nafion 115.

Note. In this document, “indirect HFR” refers to an apparent area-specific resistance that was estimated from the slope of the intermediate linear region of the polarization curve using $R_{app} \approx -\Delta V/\Delta j$. This approach provides an apparent resistance associated with the DC voltage–current response, but it should not be interpreted as a direct high-frequency resistance (HFR), because DC polarization data include activation, ohmic, and mass-transport contributions simultaneously. Direct HFR evaluation generally requires high-frequency AC resistance measurement or electrochemical impedance spectroscopy (EIS).

S4-1. Calculation Basis

To estimate the indirect HFR, the activation-loss-dominated low-current-density region was excluded. The common intermediate region of 200–600 mA cm^{-2} was selected because both membranes show a relatively quasi-linear voltage decrease in this range.

The apparent area-specific resistance was estimated using the following slope relationship:

$R_{app} \approx -\Delta V / \Delta j$

The current density was converted from mA cm^{-2} to A cm^{-2} as follows:

200 mA cm^{-2} = 0.2 A cm^{-2}, 600 mA cm^{-2} = 0.6 A cm^{-2}

S4-2. Approximate Voltage Values from the Graph

The following values were visually estimated from the provided polarization curves. Because the values are graph-read estimates, small deviations may occur depending on the digitization method.

Membrane	V at 200 mA cm^{-2}	V at 600 mA cm^{-2}	ΔV	Δj
PK-NC16 membrane	approx. 0.768 V	approx. 0.658 V	0.110 V	0.4 A cm^{-2}
Nafion 115	approx. 0.779 V	approx. 0.671 V	0.108 V	0.4 A cm^{-2}

S4-3. Indirect HFR Calculation

PK-NC16 membrane

R_{app} = (0.768 − 0.658) / (0.6 − 0.2)

R_{app} ≈ 0.274 Ω·cm²

Therefore, R_app ≈ 274 mΩ·cm².

Nafion 115

R_{app} = (0.779 − 0.671) / (0.6 − 0.2)

R_{app} ≈ 0.270 Ω·cm²

Therefore, R_{app} ≈ 270 mΩ·cm².

S4-4. Interpretation

The indirect resistance values estimated from the polarization curve are summarized below.

Membrane	Indirect R_{app}	Interpretation
PK-NC16 membrane	approx. 0.27 Ω·cm²	Ohmic-loss tendency is nearly comparable to Nafion 115.
Nafion 115	approx. 0.27 Ω·cm²	Stable reference membrane behavior in the selected linear region.

The difference between the two values is approximately 0.004 Ω·cm², or about 4 mΩ·cm², and this difference is very small and may fall within the experimental uncertainty. Therefore, based only on the polarization curves, the PK-NC16 membrane can be interpreted as exhibiting ohmic polarization behavior comparable to that of Nafion 115.

In addition, the PK-NC16 curve extends to a higher current density region up to approximately 1200 mA cm^{-2}. The voltage does not collapse abruptly after 600 mA cm^{-2}, suggesting that the

MEA maintains relatively stable operation at higher current densities. However, this high-current behavior should not be interpreted as pure HFR performance, because membrane resistance, electrode kinetics, gas diffusion, water management, and mass-transport losses are all reflected in the polarization curve [5,6].

Accordingly, the calculated R_{app} values should be treated as indirect or semi-quantitative indicators of ohmic loss, not as direct HFR measurements. Accurate HFR evaluation requires direct electrochemical diagnostic methods such as electrochemical impedance spectroscopy, high-frequency AC resistance measurement, or the current-interrupt method [7.8].

References

[1] Hwang, S. Y. et al., Synthesis of Highly Durable Sulfonated Polyketone Fibers by Direct Sulfonation Reaction and Their Adsorption Properties for Heavy Metals, Macromolecular Research, 28, 336–342, 2020. DOI: 10.1007/s13233-020-8046-8

[2] Al Lafi, A. G. et al., *Determination of the degree of sulfonation in cross-linked and non-cross-linked Poly(ether ether ketone) using different analytical techniques*, Heliyon, 2025.

[3] Shao, Y.; Yin, G.; Wang, Z.; Gao, Y. Proton exchange membrane fuel cell from low temperature to high temperature: Material challenges. *J. Power Sources* **2007**, *167*, 235–242. https://doi.org/10.1016/j.jpowsour.2007.02.065.

[4] R. Borup et al., "Scientific Aspects of Polymer Electrolyte Fuel Cell Durability," Chemical Reviews (2007).

[5] K. R. Cooper and M. Smith, "Electrical test methods for on-line fuel cell ohmic resistance measurement," *Journal of Power Sources*, vol. 160, no. 2, pp. 1088–1095, 2006. DOI: 10.1016/j.jpowsour.2006.02.086.

[6] Scribner Associates, Inc. Advanced Fuel Cell Diagnostic Techniques for Measuring MEA Resistance. *Fuel Cell Magazine* **2005**. Available online: https://www.scribner.com/wp-content/uploads/2017/06/Scribner-on-Fuel-Cell-Test-Methods-FC-Magazine-2005.pdf (accessed on 25 April 2026).

[7] J. Choi, J. Sim, H. Oh, and K. Min, "Resistance Separation of Polymer Electrolyte Membrane Fuel Cell by Polarization Curve and Electrochemical Impedance Spectroscopy," *Energies*, vol. 14, no. 5, 1491, 2021. DOI: 10.3390/en14051491.

[8] C. Zhang, et al., "A Comparative Study of Using Polarization Curve Models in Proton Exchange Membrane Fuel Cell Degradation Analysis," *Energies*, vol. 13, no. 15, 3759, 2020.
